# Electrical conductivity of poly(3,4-ethylenedioxythiophene):*p*-toluene sulfonate films hybridized with reduced graphene oxide

**DOI:** 10.1186/1556-276X-9-644

**Published:** 2014-11-29

**Authors:** Jun-Taek Lee, Sul-Hwa Choi, Jin-Yeol Kim

**Affiliations:** 1 School of Advanced Materials Engineering, Kookmin University, Seoul 136-702, South Korea

**Keywords:** Reduced graphene oxide, Poly(3,4-ethylenedioxythiophene), Graphene-polymer hybrid, Transparent conductive electrode, Interfacial polymerization

## Abstract

Reduced graphene oxide-poly(3,4-ethylenedioxythiophene):*p*-toluene sulfonate (rGO-PEDOT:PTS) hybrid electrode films were synthesized directly on a substrate by interfacial polymerization between an oxidizing solid layer and liquid droplets of 3,4-ethylenedioxythiophene (EDOT) produced by electrospraying. The EDOT reduced the graphene oxide by donating electrons during its transformation into PEDOT:PTS, and hybrid films consisting of rGO distributed in a matrix of PEDOT:PTS were obtained. These rGO-PEDOT:PTS hybrid films showed excellent electrical conductivities as high as 1,500 S/cm and a sheet resistance of 70 Ω sq^-1^. The conductivity values are up to 50% greater than those of films containing conductive PEDOT:PTS alone. These results confirm that highly conductive rGO-PEDOT:PTS hybrid films can potentially be used as organic transparent electrodes.

## Background

Transparent conductive electrode films, such as indium-tin oxide (ITO), have recently become the focus of considerable research due to their potential applications in optoelectronic devices, such as touch screens, liquid-crystal displays, organic light-emitting diodes (OLEDs), and thin-film solar cells [1-5]. ITO has been widely utilized and is considered the most effective transparent conductive electrode material because of its relatively low resistivity (approximately 10^-4^ Ω cm) and work function, qualities that allow the injection and collection of charge carriers within semiconductors. However, ITO cracks easily during repeated use because of its brittle nature [6,7], has a low optical transmittance in the near-infrared range, has a high refractive index, and requires high processing temperatures [8-10]. The flexibility of ITO electrode films is largely limited by the brittleness of the material. The transparent electrodes made of different materials, including carbon nanotubes, graphene, and conducting polymers, are required to improve the mechanical performance of the devices. In particular, cheap, flexible, and solution-processed materials are required for use in emerging electronic devices such as flexible displays. One of the objectives of research in this field is the development of high-performance electronic devices that are made entirely of plastic. However, before such technology can be realized, it is necessary to produce organic electrode materials with conductivities and stabilities comparable to those of ITO.

**Figure 1:**
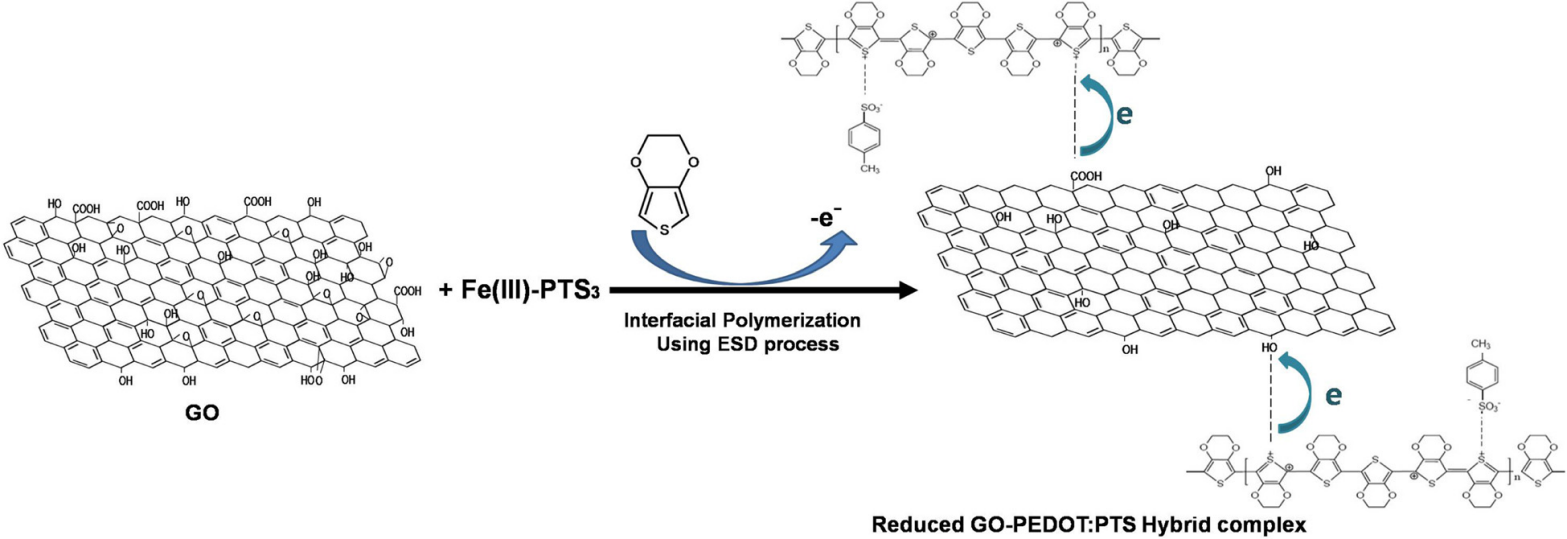
**Reaction scheme for the synthesis of a rGO-PEDOT:PTS hybrid film.** Interfacial polymerization was used between EDOT and GO in the presence of iron(III) tosylate.

**Figure 2:**
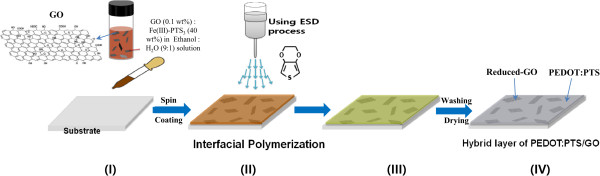
**Schematic illustration of the fabrication of a rGO-PEDOT:PTS hybrid layer on a flexible substrate. (I)** A suspension of 10 wt.% GO and 40 wt.% Fe(III)-*p*-toluene sulfonate in a 9:1 mixture of ethanol and H_2_O is spin coated on a substrate and then dried at 80°C for 3 min. **(II)** The EDOT monomer is deposited by ESD onto the GO-Fe(III)-*p*-toluene sulfonate-coated substrate. **(III)** The interfacial polymerization reaction occurs. **(IV)** After washing with ethanol and drying at 80°C for 2 min, the final rGO-PEDOT:PTS hybrid layer on a substrate film is obtained.

To find a substitute for ITO, much effort is currently being devoted to electrically conductive polymers such as poly(3,4-ethylenedioxythiophene):poly(styrenesulfonate) (PEDOT:PSS) [11,12], a kind of conducting polymer with a conjugated π-electron bonding system. PEDOT:PSS polymers have relatively good conductivity values of 500 to 1,000 S cm^-1^[13-15], good electrochemical stability, moderate transparency, and good film-forming capabilities during solution processing. But some properties of these conductive polymers, such as the conductivity, transparency, and thermal stability, have not yet attained values comparable to those of ITO and required for their use in electronic devices. In previous publications [16,17], we reported highly conductive vapor phase-polymerized poly(3,4-ethylenedioxythiophene):*p*-toluene sulfonate (PEDOT:PTS) films with conductivities as high as 1,000 to 1,100 S cm^-1^ and transmittance up to 85% in OLED and liquid crystal devices. However, these conductivities are well below that of typical amorphous ITO (≈4,000 S cm^-1^), and the transmittance of the metallic PEDOT films was generally lower than that of ITO films.In this work, we investigated the novel PEDOT:PTS films hybridized with reduced graphene oxide (rGO) for use in transparent PEDOT:PTS electrode films with enhanced electrical performance and light transmittance. The films were directly synthesized by an interfacial polymerization method (and the chemical structure of the rGO-PEDOT:PTS hybrid complex is shown in Figure 1). The structure of these hybrid films includes an rGO sheet, forming a direct connection between the PEDOT:PTS polymer chains. These hybrid electrode materials were synthesized by interfacial polymerization on an oxidizing solid layer of iron(III) tosylate on which graphene oxide (GO) was dispersed, pre-depositing this material onto a flexible substrate and treating it with liquid droplets of the 3,4-ethylenedioxythiophene (EDOT) monomer with a size of 50 to 200 nm, produced using electrospray deposition (ESD), as shown in Figure 2.

GO has a two-dimensional graphitic structure featuring a variety of chemically reactive functionalities, such as hydroxy and epoxy groups on the basal plane and carboxylic acid groups along the sheet edge [18,19], which can be differentially functionalized [20,21]. In spite of these surface moieties, a significant amount of the underlying sp^2^-hybridized carbon structure remains intact [22], allowing the approximately 1-nm-thick nanosheet to retain a high degree of planarity. Pristine GO itself is a near-insulator or a semiconductor with a differential conductivity between 1 and 5 × 10^-3^ S cm^-1^ at a bias voltage of 10 V. GO can be partially reduced by treating it with hydrazine hydrate, by exposing it to hydrogen plasma, or by exposure to a strong pulse of light. The conductivity of these rGO has been observed to be below 10 S cm^-1^, and its charge mobility is 2 to 200 cm^2^ V^-1^ s^-1^ for holes and 0.5 to 30 cm^2^ V^-1^ s^-1^ for electrons. However, reduction of GO readily produces agglomeration of the hydrophobic GO sheets, which have relatively high surface area. For example, Li et al. [23] fabricated films with good electrical conductivity by casting an aqueous suspension of conductive rGO sheets created by the controlled reduction of GO in an alkali solution, and Stankovich et al. [24] reported highly conductive polystyrene-graphene composite materials synthesized by reducing chemically modified GO in the presence of polystyrene. Additionally, transparent and conductive ceramic-graphene composite films were fabricated by the chemical reduction of GO in silica solutions, with hydrazine [25]. A significant enhancement in the conductivity of ceramic-graphene composite films is achieved by electron transfer between the two materials; the ceramic binds strongly to the carboxylic groups on the layered GO sheets. Recently, Amarnath et al. [26] introduced the reduction of GO using a pyrrole or thiophene, kinds of molecules having a π-conjugated bond system, as the chemical reducing agent, though the GO was not fully reduced to rGO. Since the reduction process is a key issue in the fabrication of rGO when attempting to obtain a conducting GO sheet, the conductivity of these rGO materials did not exceed 10 S cm^-1^.

To further extend the applications for GO-based materials, we report a novel and convenient method to prepare rGO sheets via the simultaneous reduction of GO sheets and polymerization of EDOT in the presence of Fe(III)-*p*-toluene sulfonate (an oxidant) and hybridization with polymerized PEDOT (the molecular structure and procedure are shown in Figures 1 and 2). The EDOT monomer was used to reduce the as-prepared GO by electron donation and oxygen consumption. According to previous reports [27,28], by applying a potential across a solution of thiophene, EDOT can be polymerized, leading to its use as an oxidant of thiophene or as a cross-coupling catalyst. GO itself is also known to have some oxidizing capacity and could also facilitate electron transfer. Finally, we obtained partially reduced GO sheets, and these rGO sheets were directly connected to the frame between the PEDOT:PTS polymer chains. Because of the hydroxyl or carboxylic acid groups of the GO, the positively charged PEDOT:PTS polymer chains cross-linked with the functional groups of GO with negative charges, acted as a relatively friendly linker, and facilitated electron transfer, between the GO sheet and the PEDOT:PTS polymer chains. In particular, the positively charged PEDOT:PTS polymer chains themselves are a conductor with a conductivity of 1,050 S cm^-1^.

However, the randomly dispersed partially reduced GO sheets were interconnected between the conducting PEDOT:PTS polymer chains, and the rGO sheets formed a direct bridge between the conducting PEDOT:PTS chains. As shown in Figure 2, these rGO-PEDOT:PTS hybrid thin films could then be obtained directly on a substrate. These hybrid films exhibited up to 50% better conductivity, 1,500 S cm^-1^, than layers containing conductive PEDOT:PTS alone (1,050 S cm^-1^), as well as a higher carrier density due to the effective percolation in electrical connectivity. Additionally, because the carrier density of the PEDOT:PTS chains increased significantly with the introduction of the rGOs, the conductivity of PEDOT:PTS was thereby enhanced. These results confirmed that highly conductive hybrid films can be used as effective organic transparent electrodes, opening up the possibility of new applications.

## Methods

GO (2 mg mL^-1^, dispersed in H_2_O), Fe(III)-*p*-toluene sulfonate, ethyl alcohol, and EDOT were purchased from Sigma-Aldrich (St. Louis, MO, USA) and used without further purification.

The rGO-PEDOT:PTS hybrid layered film was obtained using the procedure illustrated in Figure 2. First, the GO was re-dispersed to various concentrations in the range of 0.05 to 1 mg mL^-1^ in a 1:9 H_2_O/ethyl alcohol solution by stirring at room temperature, and Fe(III)-*p*-toluene sulfonate (for use as an oxidant) was added into the GO dispersion solution to a weight ratio of 40%. In the second step, this mixed solution was spin coated onto clean polymer substrate films and dried in an oven at 80°C for 3 min. This GO/oxidant pre-treated film was placed on the stage of the ESD system. Next, the EDOT monomer was loaded into the 10-mL syringe (equipped with a 23-G dual concentric metal nozzle) of the ESD system. The distance between the solution-loaded syringe tip and the substrate was maintained at 3.3 cm, and the ESD voltage was set to 15 kV. Using a syringe pump, the EDOT solution was injected at a rate of 50 μL min^-1^. Liquid droplets of the EDOT solution were then sprayed from the nozzle, producing droplets with sizes of 50 to 200 nm. The temperature of the ESD plate was 100°C. The polymerization of the EDOT and the reduction of the GO occurred simultaneously during the spraying of the fine EDOT droplets onto the solid GO/oxidant pre-treated substrate, as shown in Figure 2. After the polymerization process, pure conductive PEDOT:PTS layers hybridized with GO were obtained by washing with methanol to remove impurities and unreacted materials. Thus, the hybrid layered film of rGO irregularly distributed in a PEDOT:PTS matrix was directly assembled onto a substrate film.

The surface morphology and conductivity of the rGO-PEDOT:PTS films were investigated using scanning electron microscopy (SEM; JEOL JSM-633 F, JEOL, Akishima, Tokyo, Japan), atomic force microscopy (AFM; Nanoscope IIIa, Digital Instruments, Tonawanda, NY, USA), and the standard four point-probe technique (Mitsubishi Chemical Loresta-GP, Tokyo, Japan). The thicknesses of the films were measured using an ellipsometer (SE-800, SENTECH, Johannesburg, South Africa). Optical and structural analyses were carried out using ultraviolet and visible spectroscopy (UV–vis; Shimadzu UV3150, Shimadzu, Kyoto, Japan), and Raman spectroscopy (Horiba, LabRamRH, Kyoto, Japan) with a 514.5-nm excited argon laser.

## Results and discussion

**Figure 3:**
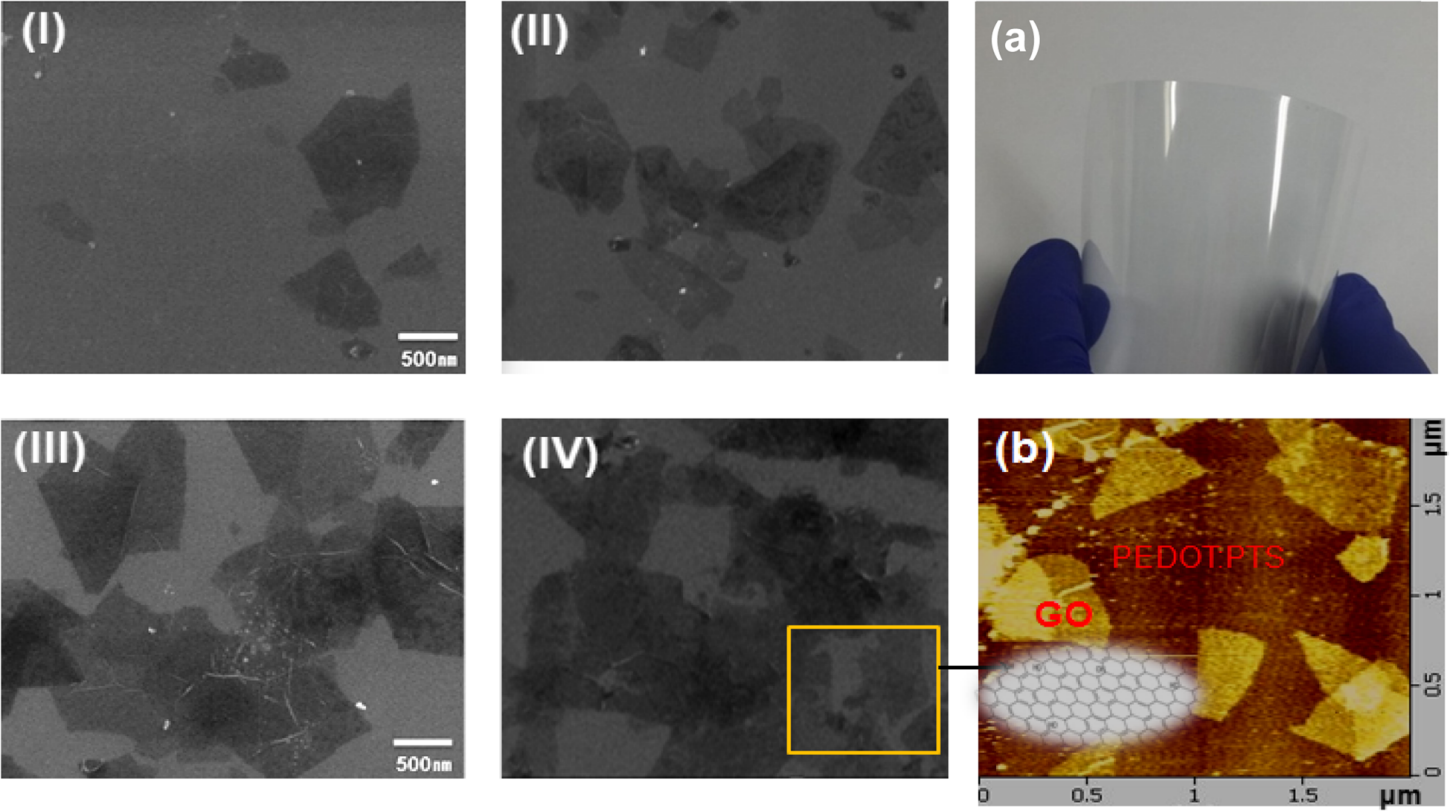
**SEM images showing the morphologies of the hybrid films.** The films were blended at rGO-PEDOT:PTS ratio by weight of **(I)** 0.005:1, **(II)** 0.010:1, **(III)** 0.025:1, and **(IV)** 0.050:1. **(a)** A photograph (100 × 100 mm) and **(b)** the AFM image of the hybrid film blended at a ratio of 0.05:1.

**Figure 4:**
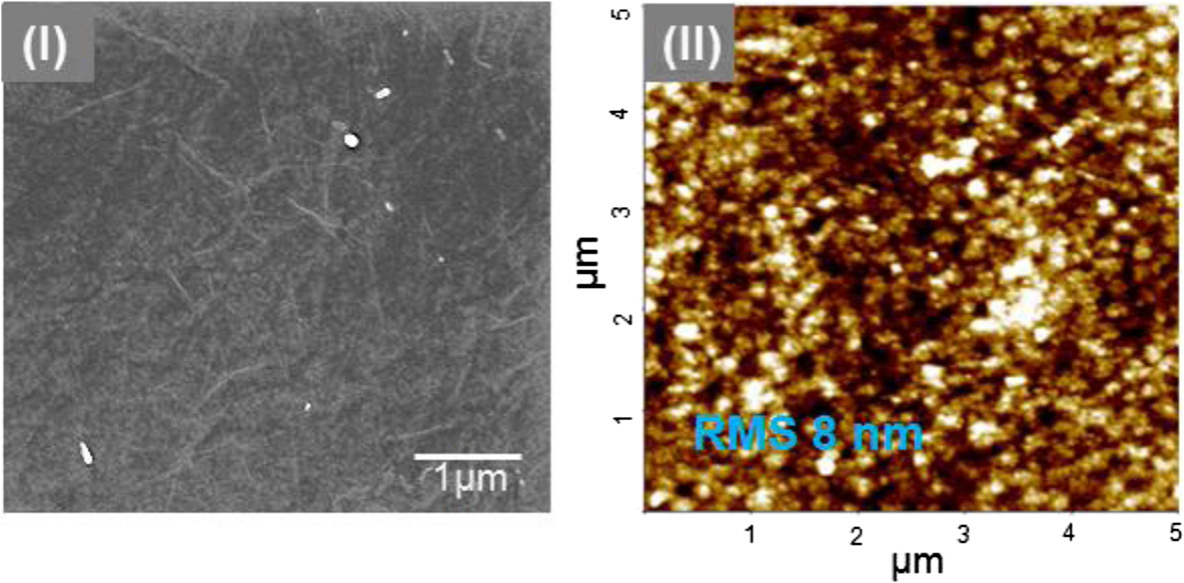
**SEM (I) and AFM (II) images of the 80-nm-thick hybrid films blended at rGO-PEDOT:PTS.** Ratio of 0.050:1.

The hybrid film, consisting of rGO irregularly embedded in the PEDOT:PTS matrix, was then directly assembled onto a substrate by electrospraying, as shown in Figure 2. The PEDOT:PTS films hybridized with rGO are obtained by interfacial polymerization between an Fe(III)-*p*-toluene sulfonate oxidizing solid layer and liquid droplets of EDOT produced by electrospraying. The deposition method using electrospraying is characterized as capable of uniformly coating a large area and highly uniform deposition on the SEM and AFM images of the rGO sheets dispersed at various concentrations into the PEDOT:PTS matrix are shown in Figure 3. Figure 3 (I-IV) shows SEM images of the near monolayer thin films of rGO hybridized with PEDOT:PTS in ratios of 0.005:1, 0.010:1, 0.025:1, and 0.05:1 (*w*/*w*), respectively. Figure 3a shows a macroscopic picture of the film sample of 100 × 100 mm size with a rGO loading of 0.05 wt.%, and Figure 3b shows an AFM image of the same sample. As revealed by the SEM and AFM images, the rGO-PEDOT:PTS hybrid films were composed of rGO sheets embedded in a conductive PEDOT:PTS matrix layer. The resulting rGO sheets 0.5 to 1 μm in size were mostly single layer, and the randomly dispersed rGO sheets were interconnected between polymerized conductive PEDOT:PTS chains. Moreover, when the rGO content was below 0.025% (*w*/*w*), no aggregation was observed, but some aggregation, manifested as overlap between rGO sheets, occurred at concentrations of 0.050% (*w*/*w*) as shown in Figure 3 (IV). Figure 4 shows the SEM and AFM images of the 80-nm-thick films of rGO hybridized with PEDOT:PTS in ratios of 0.05:1 (*w*/*w*). As shown in Figure 4, their surface condition showed a very uniform morphology. Then, RMS was 8 nm when the deposited film thickness was 80 nm.

**Figure 5:**
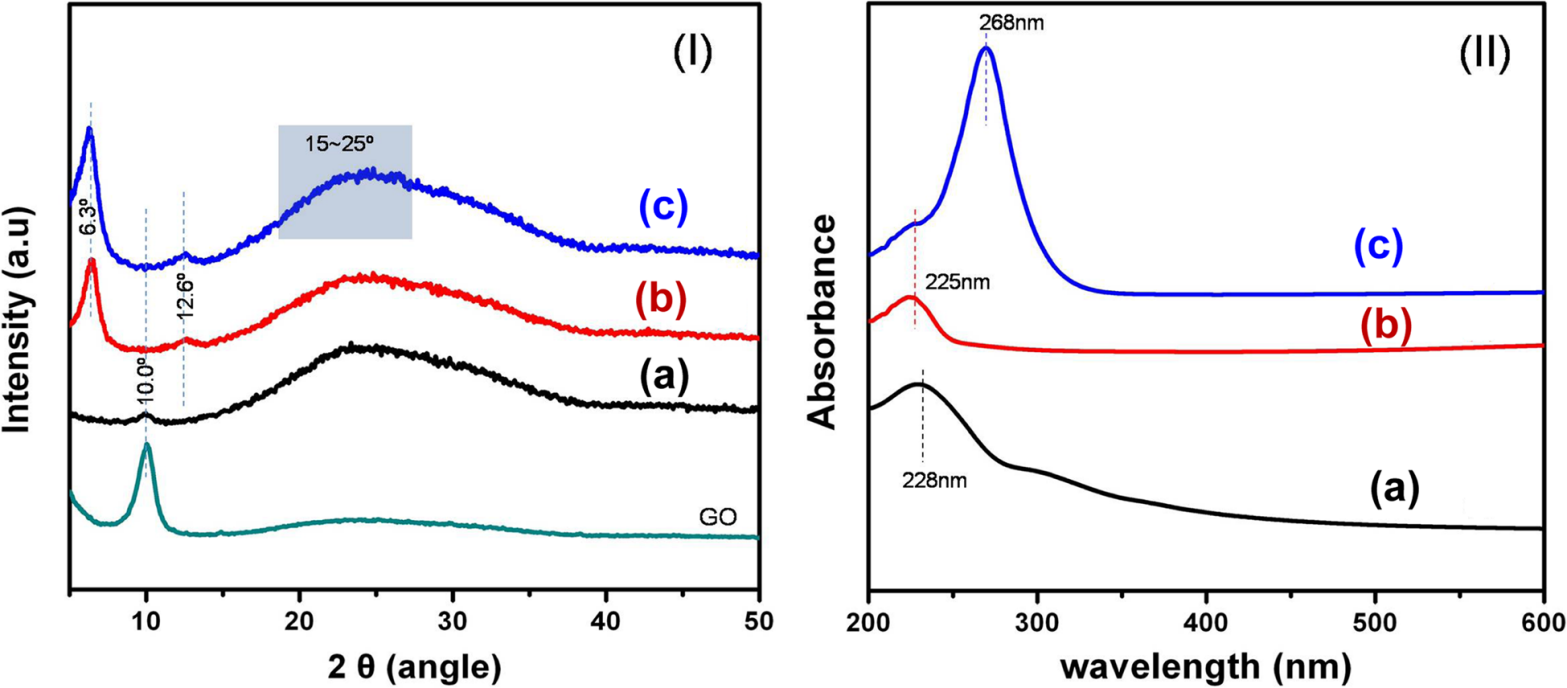
**Characteristic (I) XRD patterns and (II) UV–vis absorption spectra of the GO-based materials.** The XRD patterns of a pure GO film [curve (a)], a PEDOT:PTS film [curve (b)], and rGO-PEDOT:PTS hybrid film blended with 0.025% (*w*/*w*) GO [curve (c)] are shown alongside that of the as-prepared GO powder. The UV–vis absorption spectra include a GO film [curve (a)], a PEDOT:PTS film [curve (b)], and rGO-PEDOT:PTS hybrid film blended with 0.025% (*w*/*w*) rGO [curve (c)].

The formation of rGO-PEDOT:PTS hybrid films and the reduction of GO during polymerization were confirmed by Raman spectroscopy, X-ray diffraction (XRD), and UV–vis spectroscopy analysis. The EDOT monomer reacted with GO to produce rGO during the course of polymerization. Thus, in the reaction mixtures, the GO sheets acquired electrons during the formation of polymerized and oxidized EDOT. Some studies on the reduction of GO using chemicals such as thiophene with π-conjugated bond systems have been performed [26-28]. The XRD and UV–vis spectroscopy data required to analyze the transformation of GO into rGO by EDOT reduction are in Figure 5 (I and II). The XRD data show that there were diffraction peaks of the as-prepared GO and 0.025% (*w*/*w*) GO at 2*θ* = 10.0°, indicating that the graphite was fully oxidized into GO; this is consistent with previous data [29]. Usually, the XRD pattern of the as-prepared GO shows a peak near 10°. However, in the case of a GO sample, GO hybridized with PEDOT:PTS [Figure 5 (I), curve (c)], the XRD pattern showed a broad peak in the range of 15° to 25°, suggesting that the GO was well reduced. The characteristic GO peak near 10° disappeared. This shift in the XRD pattern, from a sharp peak at 10.0° to a broad peak at 15° to 25°, was interpreted as the result of the reduction of GO to rGO.

Figure 5 (II) shows the UV–vis spectra of a 0.025% (*w*/*w*) GO solution, a sample of GO hybridized with the PEDOT:PTS during the process of EDOT polymerization, and a sample PEDOT:PTS film, for comparison purposes. As indicated by curve (a) in Figure 5 (II), the UV–vis absorption spectrum of pure GO possessed a characteristic absorption peak at 228 nm. On the other hand, a sample of GO hybridized with the PEDOT:PTS during EDOT polymerization [Figure 5 (II), curve (c)] had a strong absorption peak at 268 nm, and no GO absorption peak at 228 nm. Additionally, the total absorption across the entire UV-visible range increased significantly, upon chemical reduction of GO. The disappearance of the characteristic GO peak at 228 nm suggested that the electronic conjugation within GO sheets was restored after the reaction. This is another indication that GO is changed into rGO during polymerization/oxidization of the EDOT monomer. According to previous reports [23,30], the GO peak at 228 nm shifts to longer wavelengths (268 nm) upon the reduction of GO. However, we were able to show the evidence about reduction of GO by EDOT, as one of the thiophene derivatives, from XRD and UV–vis spectral analysis.

**Figure 6:**
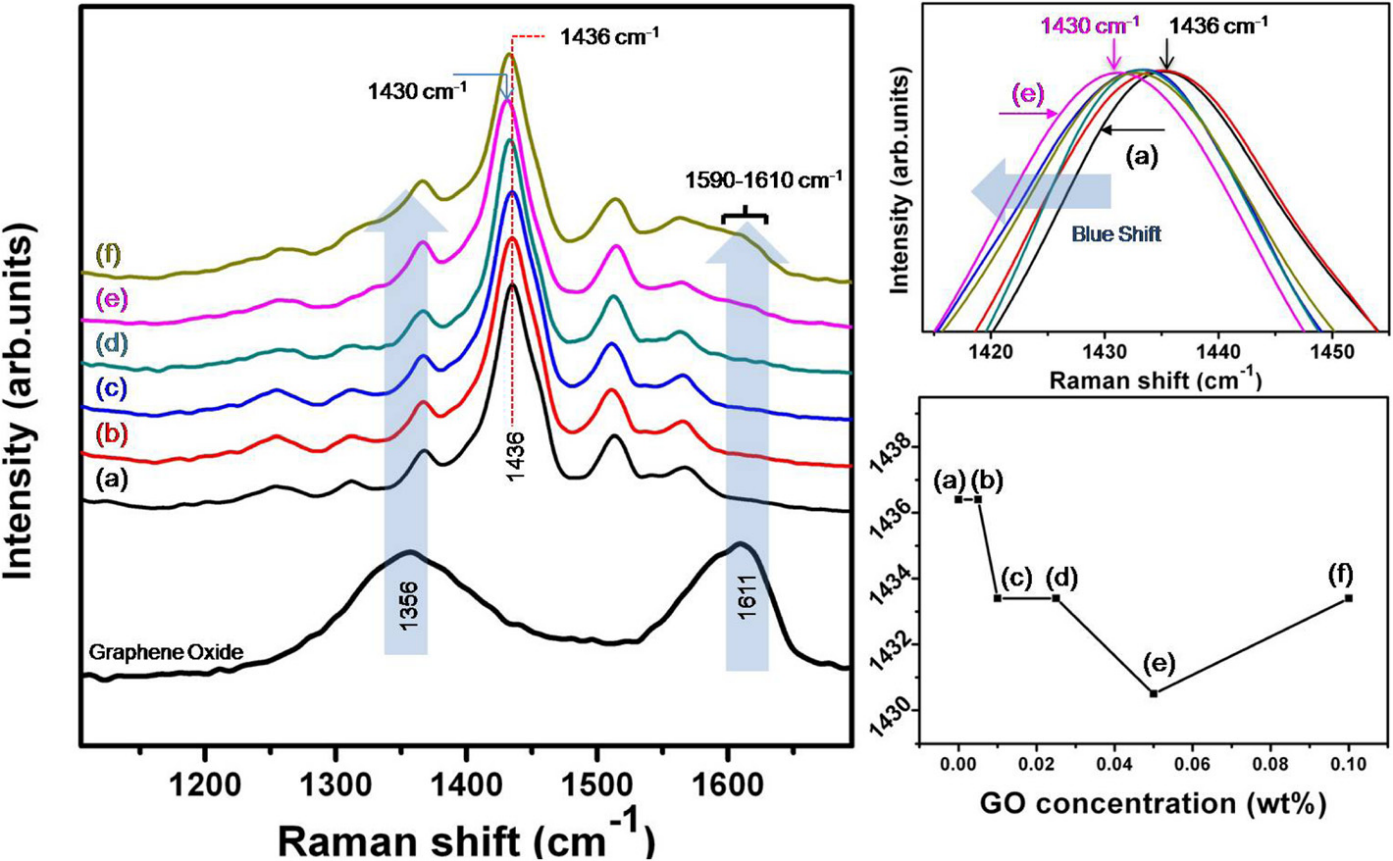
**Raman spectra of GO and rGO-PEDOT:PTS hybrid films and position change in the C = C stretching band. (I)** Raman spectra of GO and rGO-PEDOT:PTS hybrid films blended at a ratio of (a) 0.00:1, (b) 0.005:1, (c) 0.010:1, (d) 0.025:1, (e) 0.050:1, and (f) 0.100:1. **(II**, **III)** The position change in the characteristic strong C = C stretching band (1,436 cm^-1^) of the PEDOT:PTS molecule with rGO addition; the PEDOT:PTS peak at 1,436 cm^-1^ is blueshifted to 1,430 cm^-1^ when 0.05% (*w*/*w*) rGO is added. The peak position was small shifted toward longer wavenumbers at the higher rGO content of 0.10% (*w*/*w*).

That the rGO was hybridized covalently by PEDOT:PTS can be confirmed by the Raman spectra in Figure 6. Raman spectroscopy is an efficient technique for characterizing the structure and quality of carbon materials and investigating the defect structure of graphene. We also determined the degree of hybridization of the rGO with PEDOT:PTS using a Raman analysis. As shown in Figure 6, the Raman spectrum of GO shows two characteristic strong peaks at approximately 1,356 cm^-1^ (the D-band) and 1,611 cm^-1^ (the G-band). The G-band is assigned to the tangential mode and is related to the graphite E_2g_ symmetry. To further elucidate the mechanism of hybridization in the hybrid structure, we also analyzed the Raman spectra of the PEDOT:PTS and the PEDOT:PTS hybridized with rGO at the various concentrations. For the pure PEDOT:PTS film [Figure 6 (I), curve (a)], the characteristic Raman bands appeared at approximately 1,560 to 1,510 cm^-1^ (weak), 1,436 cm^-1^ (strong), and 1,365 cm^-1^ (weak) and are related to C = C stretching and C-C stretching.

In particular, the 1,436 cm^-1^ C = C stretching band showed very strong vibration. In the case of the PEDOT:PTS blended with GO at loadings of 0.005%, 0.010%, 0.025%, 0.050%, and 0.10% (*w*/*w*), the positions of the C = C stretching band changed with the GO content, as shown in Figure 6 (I), curves (b-f). Their spectra also show the combined bands related to the GO and PEDOT:PTS. First, the G-band intensity (*I*_G_) of the GO became weaker, and the peak positions were blueshifted from 1,611 cm^-1^ to 1,590-1,610 cm^-1^, upon blending with PEDOT:PTS. This is due to the reduction of GO to rGO. In this regard, Stankovich et al. [31] reported that the as-prepared rGO sample had a G-band at 1,585 cm^-1^ and a D-band at 1,354 cm^-1^, and the *I*_D_/*I*_G_ ratio increased after chemical reduction of the rGO. Similarly, in our study, the *I*_D_/*I*_G_ ratio and G-band position of the as-prepared hybrid GO changed significantly in comparison to the results reported in the literature [31].

Second, the position of the characteristic strong C = C stretching band (1,436 cm^-1^) of the PEDOT:PTS molecule was blueshifted in the peak position from 1,436 to 1,430 cm^-1^ upon blending with rGO, even though only a small amount [0.05% (*w*/*w*)] was used, as shown in Figure 6 (I), curve (e). On the other hand, their peak position was slightly shifted toward longer wavenumbers at the highest rGO content of 0.10% (*w*/*w*). As a result, a significant downward shift in the C = C stretching band vibration in the cyclic ring of PEDOT indicates that the thiophene structure in PEDOT chains changed from benzoid-rich to quinoid-rich with the introduction of rGO, whereas rGO sheets act as charged electron donors in PEDOT chains. This further confirmed that the PEDOT chains were connected to the sheets of rGO. The benzoid structure was converted to the quinoid structure so as to be more favorable to inter- and intra-chain charge transport in PEDOT. However, the presence of the quinoid structure meant that the conjugated system of thiophene molecules had delocalized electron states, which were assigned to positively charged states, generated from the benzoid structure by the influx of electrons or carriers from the rGO sheets. Therefore, the quinoid-rich structure is the typical case for electron-rich or highly doped PEDOT chains, and the observed Raman shifts may arise from a change in the structure. In this regard, Quyang et al. [32] reported that the conformational changes of the PEDOT chains from the benzoid to quinoid configuration increased inter-chain interactions, thereby enhancing the conductivity of the PEDOT polymer. This result is consistent with the increase in conductivity because the rGO sheets acted as electron donors to PEDOT chains.

The thickness of the hybrid films could be varied by varying the thickness of the GO/oxidant layer (in the first step) and EDOT monomer deposition time (in the second step) of the process. Since the hybrid films consisted of conductive PEDOT:PTS chains interconnected with conductive rGO sheets, the density of the rGO seemed to greatly affect the overall conductivity of the film. Introducing larger rGO sheets into the hybrid film increases the contact area between the polymer chains and the rGO, which results in an improvement of the charge transfer process. The electrical properties and mobility are very important because many potential applications rely on electrical behavior. Conjugate polymers, in particular PEDOT, can be straightforwardly prepared by several methods, and their electronic behavior can be reversibly changed between insulating and conducting states by redox reactions. Conductivity is the product of two important factors: the number of carrier electrons or holes and the carrier mobility, which in a loose sense is the case in which a carrier moves through a material. The electrical conductivities of most conductive polymers are in the same range as those of inorganic semiconductors or metal conductors of low quality, but there are also differences related to the purity, doping level, chain length, and defect concentration. In this work, we obtained rGO-PEDOT:PTS hybrid materials composed of conductive rGO embedded into the conductive PEDOT:PTS matrix that had an effective conductivity with high carrier density. The conductivity of these hybrid films can be enhanced considerably through charge carrier transport between the metallic, conductive PEDOT:PTS chains and the rGO, which aid in charge transport by hopping. This charge transfer, in turn, results in the increased electrical conductivity of the metallic PEDOT:PTS polymer through the effective pinning of the Fermi level inside the valence band with charge carrier inflow from the rGO.

**Figure 7:**
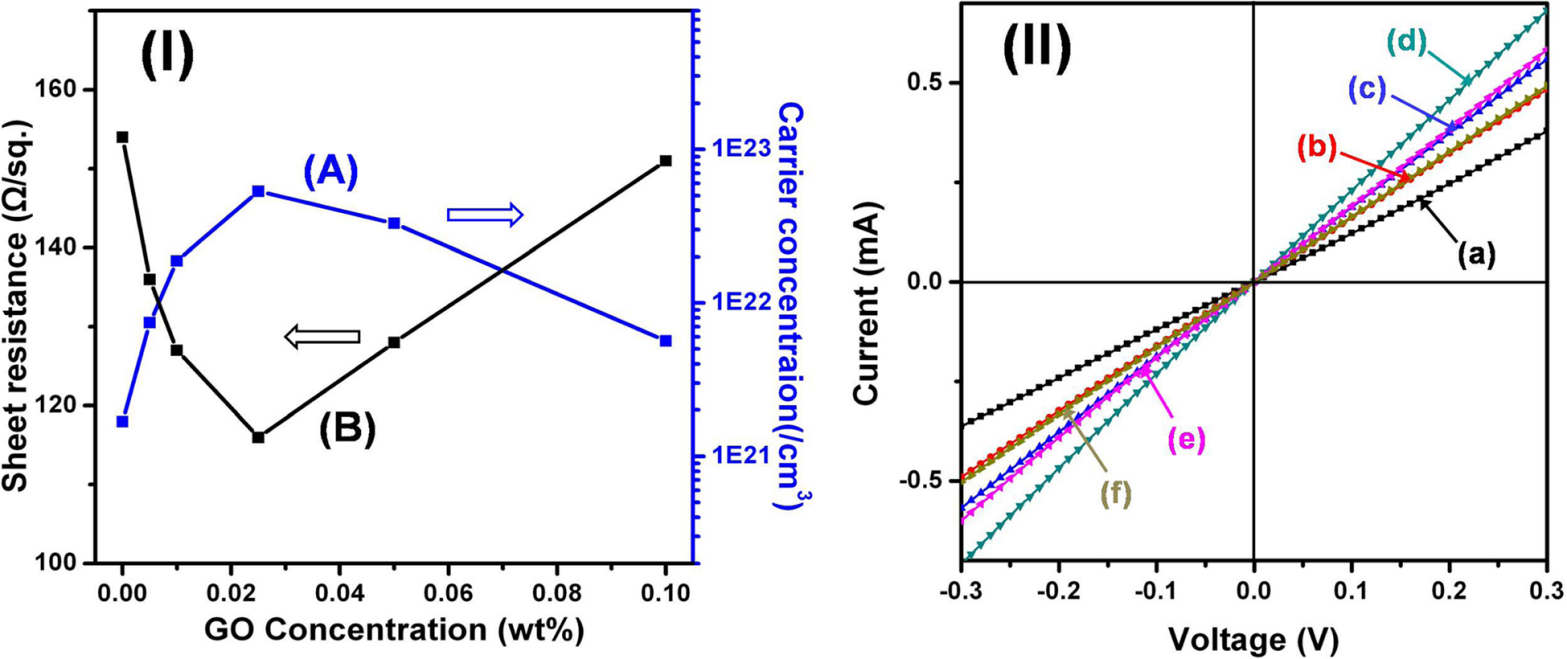
**Carrier concentration and sheet resistance, and *I*-*V *characteristics. (I)** Changes in carrier concentration and sheet resistance with the concentration of rGO in rGO-PEDOT:PTS hybrid films. **(II)***I*-*V* characteristics of the rGO-PEDOT:PTS hybrid films with rGO contents of (a) 0.000%, (b) 0.005%, (c) 0.010%, (d) 0.025%, (e) 0.050%, and (f) 0.100% (*w*/*w*).

The data in Figure 7 (I) show the sheet resistance and carrier concentration as a function of the rGO loading in the rGO-PEDOT:PTS hybrid films. The decrease in sheet resistance was related to the rGO concentration. The lowest sheet resistance occurred at a rGO concentration of 0.025% (*w*/*w*). On the other hand, concentrations greater than 0.025% (*w*/*w*) did not result in a greater decrease in the sheet resistance; instead, a slight increase was observed. Thus, the 0.025:1 rGO-PEDOT:PTS film sample had a low sheet resistance of 70 Ω sq^-1^ at an optimized thickness. These sheet resistance values gave up to 50% better conductivity than for films containing PEDOT:PTS alone. Additionally, as the rGO sheet concentration increased, the carrier concentration in rGO-PEDOT:PTS hybrid films increased by more than 1 order of magnitude from 1.68 × 10^21^ to 5.33 × 10^22^ cm^-3^. On the other hand, the carrier concentration decreased at rGO concentrations greater than 0.050% (*w*/*w*). At rGO concentrations greater than 0.050% (*w*/*w*), the increase in the sheet resistance and the decrease in the carrier concentration were due to the reduced hybrid effect resulting from some aggregation with overlap between rGO sheets [cf. Figure 3 (IV)].

Overall, all hybrid films showed some improvement in conductivity compared to PEDOT:PTS alone, which suggests a low contact resistance between elements. To evaluate the conductivity as a function of rGO concentration, a method employing a scanning probe microscope (SPM) was used. In this measurement, an SPM equipped with a diamond-tip cantilever was used to measure the electrical conductivity of six different hybrid films. The electrical contact on the other side of the film was fabricated with a conducting gold paste. Figure 7 (II) shows the differences in the current–voltage (*I*-*V*) characteristics of the six hybrid films. All *I*-*V* curves exhibited exponential increases until they saturated at the full-scale current of 0.7 mA. These curves represent typical *I*-*V* traces obtained reproducibly, depending on the electrical states of the films. As a result, PEDOT:PTS films hybridized with rGO showed a greater current response with increasing bias than the pure PEDOT:PTS film [Figure 7 (II), curve (a)]. The greatest current response was observed in the sample with a rGO concentration of 0.025% (*w*/*w*), as shown in Figure 7 (II), curve (d). On the other hand, in the samples with rGO loadings of 0.05% and 0.1% (*w*/*w*), the current response was reduced, the same trend present in the behavior of the carrier concentration/sheet resistance shown in Figure 7 (I).

**Figure 8:**
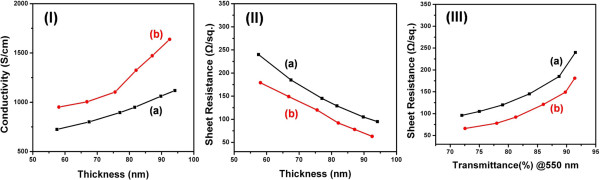
**Changes in conductivity and sheet resistance.** Changes in **(I)** conductivity and **(II)** sheet resistance with the thickness, and **(III)** changes in the sheet resistance with the optical transmittance (PET film base at 550 nm): (a) pure PEDOT:PTS film and (b) rGO-PEDOT:PTS hybrid film with a 0.025% (*w*/*w*) content of rGO.

Furthermore, these PEDOT:PTS films hybridized with rGO sheets consistently exceeded the conductivity of pure rGO sheets or PEDOT:PTS alone by more than 50% at a similar optical transparency with respect to the film thickness, as shown in Figure 8. As shown in Figure 7, the lowest sheet resistance (or highest carrier concentration) occurred at an rGO concentration of 0.025% (*w*/*w*). Concentrations greater than 0.025% (*w*/*w*) did not result in a continuous decrease in the sheet resistance; instead, a slight increase was observed. Thus, the rGO-PEDOT:PTS hybrid films blended with rGO of 0.025% (*w*/*w*) had a low sheet resistance of 70 Ω sq^-1^ with a transmittance of 80% at an optimized thickness. Figure 8 (I) shows the variation in the conductivity of the rGO-PEDOT:PTS film hybridized with rGO of 0.025% (*w*/*w*) as a function of film thickness. Four-point-probe dc measurements were performed on the pure PEDOT:PTS film and rGO-PEDOT:PTS hybrid film with a rGO content of 0.025% (*w*/*w*), and their conductivities (*σ*) were measured as a function of film thickness (*t*). The value of *σ* for the conventional pure PEDOT:PTS films was 1,050 S cm^-1^ at a thickness of 90 nm; the conductivity of rGO-PEDOT:PTS hybrid films was measured to 1,500 S cm^-1^ at the same thickness. Then, the conductivity value of rGO itself has been observed to be below 1 S cm^-1^.

The sheet resistance can be converted to electrical conductivity according to *σ* = 1/*R*_s_*t*. The value of *σ* for the rGO-PEDOT:PTS hybrid film was 1,500 S cm^-1^, which corresponds to an equivalent sheet resistance of 70 Ω sq^-1^ with 80% transmittance. The sheet resistance of the PEDOT:PTS hybrid films blended with rGO to a loading of 0.025% (*w*/*w*) [Figure 8 (II), curve (b)] was significantly decreased as thickness increased. The sheet resistance was as low as a 70 Ω sq^-1^, and an optical transmittance of 80% was obtained. The changes in sheet resistance and transmittance of the sample films are shown in Figure 8 (III). However, the conductivity of the hybrid film is 50% greater than that of the metallic PEDOT:PTS film alone. This improvement in the conductivity of the PEDOT:PTS films hybridized with rGO sheets is thought to be due to doping effects and the hybridization effect through inflow of charge carriers from rGO.

Overall, it was observed that all the rGO-PEDOT:PTS hybrid films showed improved conductivity compared to PEDOT:PTS alone and that the conductivity is largely influenced by the density of the rGO sheets. This improvement in conductivity indicated the depletion of electrons through carrier doping, resulting in a downward shift of the Fermi level of PEDOT:PTS. The work function was thereby increased, and the electrical conductivity was enhanced, resulting in the metallization of the conductive polymer through the effective pinning of the Fermi level by charge carrier inflow from the rGO. This charge transfer, together with the optimization of the hybridizing conditions and rGO sheet density, results in the enhanced electrical conductivity of the PEDOT:PTS.

## Conclusions

In conclusion, this study examined metallic conducting polymer/rGO hybrid films synthesized by interfacial polymerization between an oxidizing solid layer of iron(III) tosylate coated with GO and a 3,4-ethylenedioxythiophene (EDOT) monomer. We also report a new method for the preparation and hybridization of rGO sheets through chemical reduction in the presence of EDOT during the generation of poly(3,4-ethylenedioxythiophene) (PEDOT). These hybridized films had conductivities that were more than 50% higher than that of PEDOT:PTS alone, produced by the interconnection of rGO sheets. The optimized rGO-PEDOT:PTS films had an excellent electrical conductivity of 1,500 S cm^-1^, a sheet resistance of 70 Ω sq^-1^, and 80% optical transmittance, despite the conductivity of PEDOT:PTS itself being 1,050 S cm^-1^. Compared to the traditional compositing technique and PEDOT:PTS film-forming procedures, this process enhanced the carrier density via effective percolation in the electrical conductivity. These results confirm that highly conductive rGO-PEDOT:PTS hybrid films can potentially be used as transparent electrodes in flexible electronics.

## Competing interests

The authors declare that have no competing interests.

## Authors’ contributions

JTL and SHC participated in the experiment design, carried out the synthesis, tested the thin films, and helped draft the manuscript. JYK wrote the manuscript and supervised the work. All authors read and approved the final manuscript.
